# SparkDWM: a scalable design of a Data Washing Machine using Apache Spark

**DOI:** 10.3389/fdata.2024.1446071

**Published:** 2024-09-09

**Authors:** Nicholas Kofi Akortia Hagan, John R. Talburt

**Affiliations:** Department of Information Sciences, University of Arkansas at Little Rock, Little Rock, AR, United States

**Keywords:** Data Washing Machine, entity resolution, data curation, PySpark, distributed DWM, SparkDWM

## Abstract

Data volume has been one of the fast-growing assets of most real-world applications. This increases the rate of human errors such as duplication of records, misspellings, and erroneous transpositions, among other data quality issues. Entity Resolution is an ETL process that aims to resolve data inconsistencies by ensuring entities are referring to the same real-world objects. One of the main challenges of most traditional Entity Resolution systems is ensuring their scalability to meet the rising data needs. This research aims to refactor a working proof-of-concept entity resolution system called the Data Washing Machine to be highly scalable using Apache Spark distributed data processing framework. We solve the single-threaded design problem of the legacy Data Washing Machine by using PySpark's Resilient Distributed Dataset and improve the Data Washing Machine design to use intrinsic metadata information from references. We prove that our systems achieve the same results as the legacy Data Washing Machine using 18 synthetically generated datasets. We also test the scalability of our system using a variety of real-world benchmark ER datasets from a few thousand to millions. Our experimental results show that our proposed system performs better than a MapReduce-based Data Washing Machine. We also compared our system with Famer and concluded that our system can find more clusters when given optimal starting parameters for clustering.

## 1 Introduction

One of the main goals in creating every information system is to ensure every entity represents one and only one real-world object. This common assumption is often not achieved due to erroneous data imputation and missing values, among other reasons. Data management from its planning, acquisition, transformation, and disposal is termed data curation. This process can be time-consuming and often manual. Entity Resolution (ER) is a data curation process of determining whether two entities are referring to the same real-world objects or not (Talburt and Zhou, 2015). Entities in this context refer to any real-world object with a unique identity and may include equipment, employees, patients, etc. ER is synonymous with data deduplication, record linking, and entity disambiguation, and it is the foundation of many data curation processes, such as Master Data Management (MDM) and data fusion (Talburt et al., 2019).

With the growth in the volume of data over the years, the vast demand has shifted to a more efficient way to extract the high quantity of data for analysis and decision making in organizations. Data analytics technologies have evolved to help handle the fast growth rate of data. Two main big data analytics technologies used to process big data are Hadoop MapReduce (Dean and Ghemawat, 2008) and Apache Spark (Zaharia et al., 2016). The use of MapReduce and Spark in a distributed computing environment fits the problem of ER since comparing a pair of references for equivalence is independent of other pairs and can be carried out in parallel (Kolb et al., 2012).

The Data Washing Machine (DWM) is a proof-of-concept (POC) of an unsupervised ER system that uses frequency-based blocking and stopword removal to identify and cluster equivalent references. The DWM concept was birthed by Al Sarkhi and Talburt (2019a,b) and later improved and brought to live by Talburt et al. (2020). The DWM as a proof-of-concept is part of the effort to move from a traditional and supervised data curation to an unsupervised and automated data curation (Talburt et al., 2023) and has produced tremendous results since its inception. The unsupervised nature of the DWM lies on its ability to predict and use its own optimal starting parameter to cluster equivalent references (Anderson et al., 2023). A parameter file is a text file that contains system configurations used by the DWM. These configurations or parameters include but are not limited to “beta”, which is the blocking frequency parameter; “mu,” which is a linking threshold; “sigma,” which is a stopword removal threshold; “epsilon,” which is a cluster evaluation threshold; “excludeNumericTokens”, “removeDuplicateTokens”, among others.

Although the DWM POC has achieved tremendous clustering results, the original prototype design runs in a single-threaded mode and does not lend itself to parallelization. This single-threaded design of the legacy DWM poses two main challenges in an attempt to scale the prototype:

- It requires shared memory of tables and dictionaries by all phases in the system.
- Inability to process over 1 million records due to the limited memory and disk space in a single-threaded space.

We, therefore, solve the legacy DWM's unscalable design problem by using Apache Spark's RDD and solve the use of shared memory tables and dictionaries by extracting and utilizing intrinsic metadata from each reference. We use HDFS to store entity references, Apache YARN to manage computational resources such as CPU cores and memory per node on the cluster, and RDD for parallel and distributed data processing.

In this research, we make the following contributions:

- We introduce Spark Data Washing Machine (SparkDWM), a PySpark-based design of a legacy DWM. We focus on using memory to store intermediate data rather than a disk-based approach.
- We benchmark SparkDWM with the legacy DWM and proof that SparkDWM is a fully refactored, efficient version of the legacy DWM, and gets the same results as the legacy DWM.
- We compare SparkDWM with a prior MapReduce-based DWM called HadoopDWM or HDWM and show that SparkDWM has a better computational time than the MapReduce-based DWM. We also compare the linking and clustering performance of SparkDWM with another Distributed ER system called Famer.
- We finally show SparkDWM's scalability using publicly available benchmark ER datasets.

## 2 Related work

The attempts to redesign ER systems to meet the growing demands of big data is not foreign. Many works have been conducted on the design of distributed ER systems.

### 2.1 Scalable solutions in ER

In the work of Al Sarkhi and Talburt (2020), an attempt was made to refactor a rule-based ER system called OYSTER (Talburt and Zhou, 2013). In the design approach, the authors utilized frequency-based blocking and stopword removal prior to the linking process. This is made possible by the introduction of MatrixTokenizer which is a hash function that implements frequency-based blocking, and MatrixComparator which implements frequency-based stopword removal. Again, in their scalable implementation of OYSTER, tokens are used to re-create the reference after tokenization. The reformed reference then contains a reference identifier, the blocking key, and the comparison token. The authors used Hadoop MapReduce to prove that the newly refactored OYSTER is scalable. On the contrary, our proposed solution uses PySpark's memory-based data processing approach.

In prior work, we introduced a mapreduce implementation of the DWM called Hadoop Data Washing Machine (Hagan et al., 2024). Hadoop Data Washing Machine is a first step toward building a complete Distributed Data Washing Machine (DistributedDWM) using two of the most popular big data processing frameworks. HadoopDWM uses Hadoop Distributed File Systems (HDFS) as the storage framework, Apache YARN as the resource management framework, and capitalizes on the parallel nature of MapReduce for data processing. HadoopDWM is a complete refactor of the legacy DWM by mimicking the basic logic of the legacy DWM using MapReduce. One of the major challenges of HadoopDWM is it requires much disk space for reading and writing data. Our proposed solution uses a memory-based processing approach and hence has a better performance than the HadoopDWM system.

Famer (Saeedi et al., 2017; Obraczka et al., 2019) or the Fast Multi-source Entity Resolution system is a distributed clustering ER system designed for big datasets. It operates on Apache Flink to achieve high scalability and comparison of already existing cluster ER systems. Data from multiple sources is first blocked using multiple blocking techniques such as standard blocking and sorted neighborhoods to reduce the number of pairs that need to be compared. The blocked pairs are then compared for similarity, and similar pairs are clustered using the graph. One of the main distinctions between Famer and our proposed SparkDWM is that SparkDWM uses a frequency-based blocking technique to group references that need to be compared for similarity.

### 2.2 Learning-based solutions in ER

The marriage between machine learning and big data technologies has been a sort-after solution recently. One popular work of incorporating machine learning algorithms in scalable ER systems is Dedoop (Kolb et al., 2012). Dedoop efficiently translates user-defined ER configurations into a workable and scalable MapReduce job. The system offers multiple blocking functions and chooses the best function based on the input data to be processed. In Dedoop, all blocking processes occur in a mapper function, whereas linking of equivalent references happens in a reducer function. For the similarity comparison of references, Dedoop uses a set of machine learning classification libraries that classifies a pair of references as either linked or not linked. The usage of machine learning requires extensive training and learning of the modules in order to produce the best possible ER results. Another machine-learning-based ER requiring extensive training of module can be found in the work of Kolb et al. (2011). Their work uses similar processing concept as Dedoop and requires extensive training of the linking model for better linking of equivalent pairs.

Apache Flink is a well-known distributed computing technology for processing big data in parallel and used to scale ER applications. Nentwig et al. (2017) utilized Apache Flink and its graph processing API called Gelly. In Gelly graph, a set of entities represent the vertices and the links between the entities represent the edge of the graph. With this information, they construct a similarity graph and formed clusters from linked entities. The input vertices and edges are read into a Gelly graph, and a set of transformations are applied to create a cluster of pairs that were considered to be similar in nature. Intermediate data from this operation are stored on disk. The researchers also used the “TupleX” transformation in Flink to reduce the network traffic among computational nodes during complex transformations. Their approach requires data pre-processing before distributing the data in Flink to be processed.

Mostly the input data used to test ER systems are rich in other metadata that could potentially be useful in the matching process. Blast (Simonini et al., 2016) is a system designed to utilize intrinsic information from input data to improve blocking and matching results in ER. Loose schema from these references is extracted using some sort of similarity function. Token-blocking and meta-blocking are used to group references having the same blocking key, and only such references are compared for similarity.

### 2.3 Apache spark-based solutions in ER

ER has been applied extensively in the healthcare space to ensure patients records are not wrongfully classified. One of these application of ER in the healthcare space using a distributed processing framework is found in the work of Wang and Karimi (2016). Their distributed duplicate detector was built using Apache Spark to efficiently identify and cluster equivalent references using the k-nearest neighbor classifier. This work does data pre-processing using Natural Language Processing (NLP) to remove all data quality issues found in the input data. They also train part of the input dataset for better matching among references.

The work of Pita et al. (2015) outlined processes to perform record linking for healthcare data using Apache Spark. Their process first requires an extensive data quality check and identifying attributes in the data that may be more suitable for record linking. The next step is to apply the traditional ETL process to standardize and fix all data quality issues prior to loading it in Spark. Finally, Spark is used to perform record linking and the system requires a subject-matter expert to review the links formed. The researchers perform data standardization and pre-processing before the actual linking process, which is frowned upon in SparkDWM. Our proposed systems is a reversed paradigm of the traditional ETL process where we cluster first and clean next.

SparkER (Simonini et al., 2018; Gagliardelli, 2019) is another system designed to use intrinsic metadata from references to perform record blocking and linking. SparkER uses Spark for efficient clustering of equivalent references and offers a wide range of similarity comparison algorithms for linking. Prior to the similarity computation and linking, SparkER applies meta-blocking to group references having the same blocking key. In the loose schema generator, attributes are partitioned using Locality-Sensitive Hashing to group attribute values according to their similarity. To reduce the possibility of not comparing the same attributes more than once, the attributes with the highest similarity score are kept. Transitive closure is applied to the kept attributes, and all other duplicated attributes are kept in a blob partition and an entropy score is computed for all clusters formed.

### 2.4 Research gap

In this sub-section, we point out some of the gaps identified in the related works discussed in sub-Sections 2.1 and 2.2. Firstly, the works of Al Sarkhi and Talburt (2020) and Talburt and Zhou (2013) were for a rule-based supervised ER system called OYSTER. The nature of OYSTER requires metadata alignment and specification of both blocking and similarity comparison rules using data attributes prior to job execution. For instance, linking two references if the last name of one record is the same as the last name of another record. The difference between their work and our work is our work does not depend on rules for both blocking and linking but rather uses the frequency statistics of tokens in each reference to make blocking and linking decisions.

Secondly, the works of Kolb et al. (2012), Kolb et al. (2011), Wang and Karimi (2016), Nentwig et al. (2017), and Simonini et al. (2016) are all learning-based ER solutions. Just like any other machine-learning model, the learning-based design approach requires the training and learning of the model in other to produce the best linking result. Our system design approach does not require any model learning and only breaks each row of records into tokens and uses frequency statistics to group records that need to be compared and linked. Also, some of the works adopt Hadoop Mapreduce for design, which is known to have a poor performance than Apache Spark due to the constant reading and writing of data to and from disk in MapReduce.

Lastly, for the Apache Spark-based design approaches, the works either require data pre-processing or use a different blocking approach. For instance, in the work of Pita et al. (2015), the system requires data pre-processing and clear linking records. Our proposed system does not require data pre-processing nor standardization prior to linking equivalent references. Similarly in Simonini et al. (2018) and Gagliardelli (2019), they utilized a Locality-Sensitive Hashing (LSH) blocking approach to group records that need to be compared for similarity. Our proposed system, on the other hand, utilizes a frequency-based blocking approach to group records before applying a linking model.

## 3 Methodology

SparkDWM^1^ is a distributed scalable implementation of a legacy DWM using Apache Spark's Resilient Distributed Datasets (RDD).^2^ SparkDWM mimics all the basic ER processes in the legacy DWM including blocking, similarity comparison, transitive closure for form clusters, and cluster evaluation using entropy. SparkDWM is a memory-based data processing framework and an improved version of Hadoop MapReduce, which is a disk-based data processing framework. Spark is an open-source multi-language big data processing engine that runs on a cluster of computers. Spark's multi-language capability includes programming languages such as Python (PySpark), Scala (Spark), Java, and R (SparkR).

In SparkDWM, we utilized PySpark, which is an interface for Apache Spark in Python, as the programming framework to refactor the legacy DWM. PySpark is a Python API for Spark applications that allows non-Java, R, or Scala programmers to write Spark applications in Python. PySpark has support for Spark Core, which is the base execution engine for Spark and is comprised of RDD. RDD (Zaharia et al., 2012) in PySpark is an immutable, distributed collection of data elements partitioned and assigned to multiple computational nodes on a cluster for parallel processing. RDD is made up of Transformations and Actions. Transformations modify a previous RDD and save the resulting RDD in memory whereas Actions operate on an RDD to produce actual physical results.

**Figure 1:**
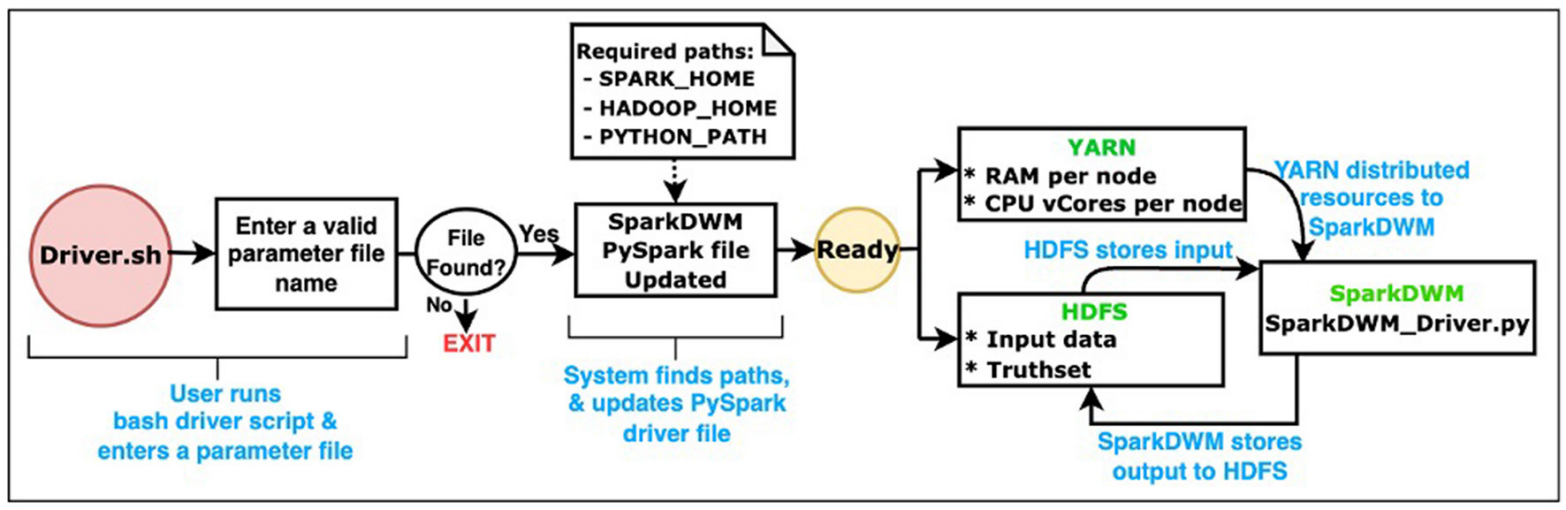
General process workflow interaction of HDFS, YARN, and SparkDWM PySpark RDD.

**Figure 2:**
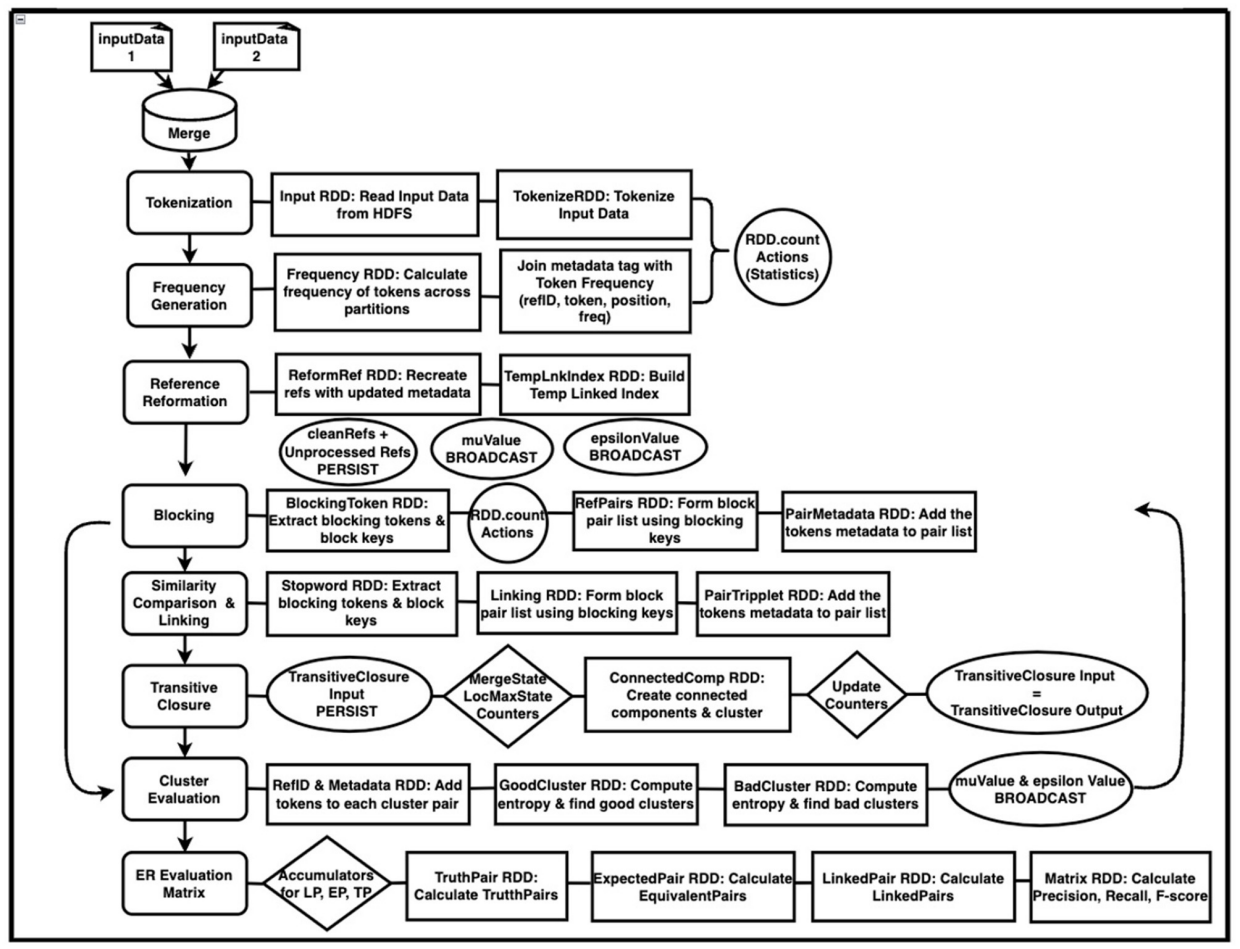
Design architecture of SparkDWM using PySpark RDD.

SparkDWM uses Hadoop Distributed File System (HDFS) as the data storage framework. HDFS (Shvachko et al., 2010) is a distributed file system designed to store large volumes of data across a cluster of computational nodes. It allows for easy scalability of a cluster to hundreds and thousands of nodes. We also utilize Apache YARN (Vavilapalli et al., 2013) as the resource management platform to equally allocate resources such as CPU cores and memory to available executors on the Spark cluster. Figure 1 below depicts the general process workflow and the interaction between HDFS, YARN, and SparkDWM. The end user runs the “Driver.sh” bash script, which prompts the user to enter a valid parameter file. A parameter file is a file that contains settings used to execute the program, and these settings are unique for each dataset. The bash script then finds some system environments such as SPARK_HOME, HADOOP_HOME, and PYTHON_HOME and updates the main SparkDWM_Driver.py script. At that point, SparkDWM is ready for resources and input dataset for execution. All CPU vCores and memory per each node are accumulated on YARN for the entire cluster and redistributed back to nodes as and when needed for SparkDWM. At the same time, the input data and the truth set file are all partitioned and stored on HDFS using a replication factor of 128 MB per block on each computational node. All the individual execution processes are displayed in Figure 2 below. SparkDWM produces two sets of outputs, a Linked Index file and a system statistics file. The Linked Index file is a file that contains only two columns: the reference identifier column and the cluster identifier column. For each reference, SparkDWM produces the cluster to which such reference belongs to. The Linked index file is stored back on HDFS and made ready for download by the end user as shown in Figure 1.

Since SparkDWM is a complete refactor of the legacy DWM, the main phases of the DWM are followed and redesigned using PySpark RDD. Figure 2 below shows the overall step-by-step design architecture of SparkDWM. SparkDWM is an iterative system comprising reference tokenization, frequency generation, reformation of references, forming blocks of references to be compared, similarity comparison of reference pairs, transitive closure to create clusters, evaluation of clusters, and computation of ER matrix to evaluate the performance of SparkDWM. Each phase in SparkDWM is explained in Sub-sections 3.1–3.8.

### 3.1 Tokenization

In SparkDWM, the merged dataset is first stored in a staging file, and the record header, which is always the first row in the reference list, is removed. Removing the reference header in the staging area ensures that none of the references are missed after partition. Partitioning the input data before removing the header will remove the first row from each partition, reducing the size of the original references. The reference in the staging file is partitioned and stored on HDFS using a replication factor of 128 MB. The input data will then be ready to be read by the first RDD in SparkDWM. The “spark context.textFile()” method is then applied to each HDFS partition of the input data to create a tuple of key-value pairs. This process is shown in the Tokenization section of Figure 2.

The tokenization process removes all unwanted characters from the references and keeps only string and numeric tokens. Unwanted characters may include all special characters that are not words or numbers. SparkDWM uses two types of tokenization functions: the tokenizer splitter and compress. The splitter removes all unwanted characters and splits by the given delimiter. In contrast, the compress tokenizer removes all unwanted characters and replaces white spaces, thereby having a compressed long token. Finally, tokenization statistics such as “tokens found”, “numericTokens”, “uniqueTokens” etc., are extracted using the “RDD.count()” function, as shown in Figure 2.

### 3.2 Frequency generation

The next step is to compute the frequency of each token from the tokenization step using the basic word count algorithm in PySpark. In the frequency generation phase, each token is mapped using a lambda function and the result from this transformation is saved to memory. The key to the map is a token, and the value is a numeric value of 1 for each token. All values belonging to each key group is then shuffled and summed up using the “reduceByKey()” method in PySpark.

Table 1 below shows output from computing token frequencies for references as well as some intrinsic token metadata in SparkDWM. The key is the first element of the parent tuple, and the second element of the parent tuple represents the value. For instance, in the output record “['AARON', (2, 'A813025′, 33)]”, the token “AARON” represents the key of the RDD, and (2, 'A813025′, 33)” represent the value. The value “(2, 'A813025′, 33)” contains the positional index of the token, which is 2, the reference identifier from which the token was found which is 'A813025′, and finally the frequency of the token which is 33. The role of the reference identifier inside the value tuple is to ensure that each token belonging to the same reference identifier is correctly captured and utilized in the reference reformation phase.

**Table 1:** Computed frequencies of tokens with other intrinsic metadata in SparkDWM.

**Token frequencies**
(‘WINSTON', (7, ‘A960838', 31)) (‘WINSTON', (7, ‘A974515', 31)) (‘WINSTON', (9, ‘A750205', 31)) (‘WINSTON', (9, ‘A942770', 31))
(‘AARON', (2, ‘A813025', 33)) (‘AARON', (2, ‘A824917', 33)) (‘AARON', (2, ‘A875214', 33))
(‘59', (12, ‘A776838', 2)) (‘59', (12, ‘A844925', 2))
(‘DONALD', (1, ‘A770538', 3)) (‘DONALD', (2, ‘A816319', 3)) (‘DONALD', (1, ‘A882820', 3))
(‘DWIGHT', (3, ‘A770538', 3)) (‘DWIGHT', (3, ‘A816319', 3)) (‘DWIGHT', (3, ‘A882820', 3))
(‘27052', (10, ‘A770538', 3)) (‘27052', (10, ‘A816319', 3)) (‘27052', (10, ‘A882820', 3))
(‘131', (11, ‘A816319', 2)) (‘131', (11, ‘A882820', 2))

In ER, because there is a higher possibility of having tokens that are the same but can be used in different contexts, we store the reference identifier for each token for easy identification. For instance, the token “Grant” may represent a person's last name in one case and might mean someone living on “Grant St”. Although similar tokens may be found after the tokenization and frequency generation, their positional index may be different. For instance, if one reference is of the form first name, last name, and another reference is of the form last name, first name, the positional index of the first name token in one reference may be different from the second reference. An example of this scenario is “output 1–['WINSTON', (7, 'A974515′, 31)]” vs. “output 2–['WINSTON', (9, 'A750205′, 31)]”. The positional index of the token “WINSTON” in output 1 is 7, whereas that of output 2 is 9.

### 3.3 Reference reformation

One of the main reasons for keeping the tokens per each reference identifier, the positional index of tokens, and reference identifier in the tokenization and frequency generation steps is to maintain the information needed to reform the references while maintaining their original integrity. The reservation of intrinsic metadata helps to eliminate the storing tokens and token frequencies in a shared dictionary, as done in the legacy DWM. Storing tokens and frequency information in a single dictionary causes out-of-memory errors and, hence, program failure when processing larger volume of data.

In the reference reformation phase, the logic for a basic word count in PySpark is used where the “RDD.map()” method is used to extract the reference identifier as the key and the intrinsic metadata as the value. The next step is to use the “RDD.reduceByKey()” method to group all metadata belonging to a particular key group. The reformed reference RDD is utilized in many subsequent stages in the process, including the Blocking and Cluster Evaluation. To recreate the reference as shown in Table 2 below, the reference identifier is pulled from the output in section 3.2 and used as a key, and the values inside each reference identifier comprise the positional index, token, and the frequency of the token. The output shown in Table 2 below shows the reformed references with the reference identifier as key and the tokens together with their positional index and frequencies as values. For instance, for the value “2: 'AADLAND^∧^ 1′”, the 2 is the positional index of the token in the reference identifier 'A944634′, AADLAND is the token, and 1 is the frequency of the token in the entire input dataset.

**Table 2:** Reformed references in SparkDWM using intrinsic metadata.

**Reformed references**
(‘A944634', {1: ‘IAN^∧^ 1', 2: ‘AADLAND^∧^ 1', 3: ‘LARS^∧^ 1', 4: ‘29021^∧^ 1', 5: ‘HIGH^∧^ 1', 6: ‘SIERRA^∧^ 1', 7: ‘TRL^∧^ 4', 8: ‘SANTA^∧^ 1', 9: ‘CLARITA^∧^ 1', 10: ‘CA^∧^ 3', 11: ‘91390^∧^ 1', 12: ‘490^∧^ 1', 13: ‘46^∧^ 1', 14: ‘2048^∧^ 1'})
(‘A755471', {1: ‘MYRA^∧^ 2', 2: ‘AARGAARD^∧^ 1', 3: ‘ESPERSEN^∧^ 2', 4: ‘1224^∧^ 2', 5: ‘MAGNOLIA^∧^ 2', 6: ‘ST^∧^ 6', 7: ‘WINSTON^∧^ 31', 8: ‘SALEM^∧^ 31', 9: ‘NC^∧^ 47', 10: ‘27103^∧^ 6', 11: ‘117^∧^ 1', 12: ‘15^∧^ 1', 13: ‘8521^∧^ 1'})
(‘A869762', {1: ‘GREGORY^∧^ 1', 2: ‘AARON^∧^ 33', 3: ‘A^∧^ 3', 4: ‘7514^∧^ 1', 5: ‘DIVALDI^∧^ 1', 6: ‘ST^∧^ 6', 7: ‘LEWISVILLE^∧^ 1', 8: ‘NC^∧^ 47', 9: ‘27023^∧^ 1', 10: ‘672^∧^ 1', 11: ‘52^∧^ 1', 12: ‘2262^∧^ 1'})
(‘A813025', {1: ‘ALLEN^∧^ 1', 2: ‘AARON^∧^ 33', 3: ‘IKAIKA^∧^ 1', 4: ‘3830^∧^ 1', 5: ‘COUNTRY^∧^ 3', 6: ‘CLUB^∧^ 3', 7: ‘RD^∧^ 13', 8: ‘J^∧^ 1', 9: ‘WINSTON^∧^ 31', 10: ‘SALEM^∧^ 31', 11: ‘NC^∧^ 47', 12: ‘27104^∧^ 10'})
(‘A844925', {1: ‘DAVIS^∧^ 2', 2: ‘AARON^∧^ 33', 3: ‘SCOTT^∧^ 2', 4: ‘3211^∧^ 4', 5: ‘KINNAMON^∧^ 4', 6: ‘RD^∧^ 13', 7: ‘WINSTON^∧^ 31', 8: ‘SALEM^∧^ 31', 9: ‘NC^∧^ 47', 10: ‘27104^∧^ 10', 11: ‘834^∧^ 2', 12: ‘59^∧^ 2', 13: ‘6144^∧^ 2'})

### 3.4 Blocking

Pairwise comparison is one of the most commonly used and acceptable ways of comparing two references for similarity. It requires that every entity reference is compared with all other references in the dataset. This process is computationally expensive and often inefficient when dealing with big data. To solve this problem, record blocking (Christen, 2012; Papadakis et al., 2015, 2020) is used to group references based on a common blocking key, and only references in a particular block are compared for similarity.

In SparkDWM, blocking begins an iteration, and references that meet the blocking condition are selected for processing. Blocking in SparkDWM is made up of 3 stages, namely the “Extraction of Blocking Tokens”, the “Creation of Blocking Keys” from the extracted tokens, and the “Block Pair Deduplication” phase. The input RDD for the extraction of blocking tokens is the reformed references. The main blocking parameter used in SparkDWM is a “beta” value. Beta represents a frequency threshold for a token to be considered a blocking token. Tokens that qualify to be blocking tokens are those with a frequency between 2 and beta. For instance, as shown in Table 3 below, extracted blocking tokens from the reference “A956423” are “LLOYD”, “AAARON”, “DEAN”, and “SPICEWOOD”. Similarly, tokens extracted from the reference “A935026” are “NENA”, “ABAI”, “IKWECHEGH”, “LOCHURST”, and “PFAFFTOWN”. The “RDD.map()” method is used to extract such tokens. After the tokens have been extracted, they are used to form blocking keys.

**Table 3:** Sample output from all blocking phases in SparkDWM.

**Blocking step**	**Sample output from SparkDWM**
Extraction of blocking tokens	(‘A956423', [‘LLOYD', ‘AAARON', ‘DEAN', 'SPICEWOOD']) (‘A921659', [‘NAOMI', ‘AUDREY', 'STRATFORD']) (‘A948701', [‘LOCHURST', 'PFAFFTOWN']) (‘A935026', [‘NENA', ‘ABAI', ‘IKWECHEGH', ‘LOCHURST', 'PFAFFTOWN']) (‘A922259', [‘NATHEN', ‘ABADIE', ‘HARRY', ‘CENTURY', ‘BLVD', 'KERNERSVILLE'])
Creation of blocking keys	(‘ESPERSENMYRA', 'A755471') (‘ESPERSENMYRA', ‘A912696') (‘MAGNOLIAMYRA', ‘A755471') (‘ESPERSENMAGNOLIA', ‘A755471') (‘CLUBCOUNTRY', ‘A813025') (‘DAVISSCOTT', ‘A844925')
Deduplication of blocking reference pairs	(‘A770538:A882820', 10) (‘A780828:A887611', 3) (‘A816319:A882820', 10) (‘A824917:A875214', 1) (‘A922259:A992523', 15)

Just as in the legacy DWM, SparkDWM has two ways of creating blocking keys, either by single tokens or by pairs of tokens. If the key creation type is to block by singles, the individual tokens from each reference represent the blocking key. However, if the blocking type is to block by pairs of tokens, pairs are formed from each reference in ascending order or magnitude. For instance, to form blocking keys from “('A921659′, ['NAOMI', 'AUDREY', 'STRATFORD'])”, the keys “NAOMIAUDREY”, “NAOMISTRATFORD”, and “AUDREYSTRATFORD” will be formed. Similarly, for “('A956423′, ['LLOYD', 'AAARON', 'DEAN', 'SPICEWOOD'])”, the keys “LLOYDAAARON”, “LLOYDDEAN”, “LLOYDSPICEWOOD”, “AAARONDEAN”, “AAARONSPICEWOOD”, and “DEANSPICEWOOD” will be formed. These formed blocking keys will serve as the key for the RDD, and the reference identifiers will represent the values. Examples of this type of key value pair output is shown in row 2 of Table 3.

The next step in the blocking phase after creating the blocking keys is to group all reference identifiers using the blocking keys. For instance, for the output ('ESPERSENMYRA', 'A755471′) and this ('ESPERSENMYRA', 'A912696′), the output will be ('A755471:A912696′, 'ESPERSENMYRA') representing block pair that need to be compared. The final step in the blocking phase is to deduplicate the created blocking pairs of references for comparison. Deduplication of blocking keys is to ensure each pair is compared only once. The third row in Table 3 below shows the results of the deduplication of blocking pairs. For instance, “('A770538:A882820′, 10)” means the pair of reference was seen 10 times during the blocking process, hence, it will compare only once.

### 3.5 Similarity comparison

All pairs of references from the block deduplication RDD are compared for similarity, and the pairs that turn out to be equivalent are linked. SparkDWM uses two very crucial parameters at this phase, namely “Sigma” and “Mu”. Sigma is another token frequency threshold for eliminating stopwords. Stopwords are tokens having a frequency higher than the given sigma threshold. It has been found that stopwords do not positively improve the linking performance of ER and sometimes may even hinder the performance negatively (Al Sarkhi and Talburt, 2019a,b). Due to this reason, they are eliminated in order to have a reduced number of tokens in each reference to compare, which further reduces the computational complexity in ER. The second parameter used for linking in SparkDWM is “mu”. Mu is a linking threshold for categorizing a pair of references as linked or not linked. The mu value is a decimal value between 0 and 1, with 0 representing the lowest similarity score and 1 representing the highest score. For instance, if a mu is set to 0.68, all pair scores up to 0.68 and above are considered linked pairs.

SparkDWM uses a similarity matrix comparator (Li et al., 2018) for linking equivalent references. The matrix comparator is a variant of the Monge Elkan comparator and uses Damerau Levenshtein Edit Distance (D-LED) for accessing the similarity of tokens. Each reference pair is passed through the similarity function using the “RDD.map()” method, and linked pairs from the resulting RDD are extracted using the “RDD.filter()” method. Similarity scores from the reference pairs are filtered for links by comparing the similarity score with the given mu threshold. The output from the similarity comparison phase includes the linked pairs, the inverse of the linked pairs, and the pair itself, which is the first item of the composite key in the linked pairs. An example of this output is shown in Table 4. This output RDD will serve as the transformation input for the transitive closure phase.

**Table 4:** Output from the similarity comparison phase of SparkDWM.

**Linked reference pairs**	**Inverse of linked pairs**	**Pair-self**
(‘A770538.A816319', ‘A816319')	(‘A816319.A770538', ‘A770538')	(‘A770538.A770538', ‘A770538')
(‘A770538.A882820', ‘A882820')	(‘A882820.A770538', ‘A770538')	(‘A770538.A770538', ‘A770538')

As shown in Table 4, the first element in the tuple before the comma in the linked reference pair column represents the pairs of references that SparkDWM considered as equivalent pairs. For instance, in “('A770538.A816319′, 'A816319′)”, the references A770538 and A816319 are similar and therefore linked. The pair then serve as the key in the tuple, and the value is the second element of the pair. This output structure is necessary and follows the accepted record structure for the CCMR algorithm used in Sub-section 3.6 below.

### 3.6 Transitive closure

**Figure 3:**
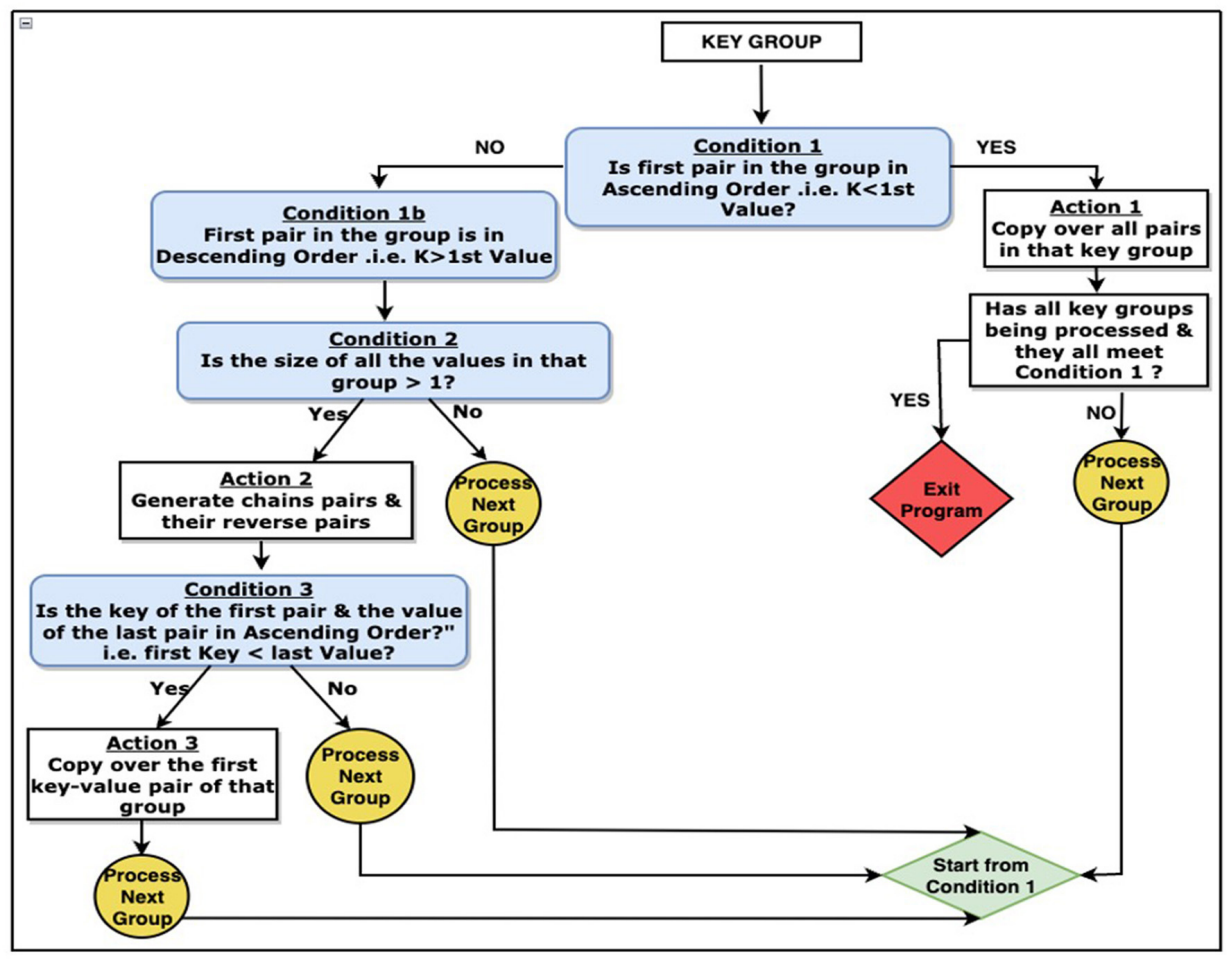
Decision tree of the CCMR transitive algorithm used in SparkDWM.

SparkDWM uses the logic from the Connected Components with MapReduce (CCMR) algorithm to find clusters of reference. CCMR algorithm uses a graph-based approach to find the connections between references where entities are represented as vertex and the similarity between entities as the edge. CCMR was first introduced by Seidl et al. (2012) and improved by Kolb et al. (2014). During the transitive closure iteration in SparkDWM, “RDD.accumulator()” method is used to store the “mergeState”, “localMaxState”, and the “clusterCount” statistics. The “mergeState” is when a pair of reference identifiers are arranged in ascending order; the opposite is true for the “localMaxState”. The accumulator values are used to determine whether the next transitive closure iteration will happen or not. The transitive closure iteration ends when the “mergeState” accumulator value is 0. Figure 3 shows step-by-step breakdown of the CCMR transitive closure algorithm used in SparkDWM. The process starts by comparing each key group in the cluster and making decisions until the exit point is met.

### 3.7 Cluster evaluation

One of the main duties of a subject matter expert in the traditional data curation process is to manually analyze and determine whether clusters formed by an ER system are good or bad clusters. This process can be cumbersome, especially when dealing with big data. One of the promising breakthroughs of the legacy DWM upon which SparkDWM was developed is the system's ability to automatically evaluate formed clusters, thereby eliminating the human-in-the-loop, a common issue in traditional data curation. This is done by computing the entropy score of clusters using a variant of Shannon Entropy to find the level of organization or disorganization between clusters. A crucial parameter utilized at this phase is the “entropy” threshold, which is a decimal value between 0 and 1, with 0 representing high disorganization and 1 representing high organization of clusters. Some RDDs used in this phase include “map()”, “union()”, “filter()”, and “join()”. The final output of the cluster evaluation phase is either good clusters or bad clusters. All good clusters are stored in a linked index file, whereas bad clusters are merged with unprocessed references from the blocking phase for the next program iteration.

### 3.8 ER matrix

The performance of most ER systems is determined using well-defined statistical measures. If a truth set file accompanied by the original dataset is loaded into SparkDWM, the truth file is copied into a staging area and loaded to HDFS for the ER matrix process. A truth set is a file that has two columns which contain each reference identifier on one column and its corresponding cluster identifier on the second column. This helps to know if the SparkDWM system performed well or not. The input for the ER matrix is all good cluster references and the truth file (if any). The “spark.sparkContext.textFile()” method is used to read the truth set from HDFS and joined with the good cluster RDDs formed. Again, the “RDD.accumulator()” method is used to aggregate the matrix statistics. A pair-counting approach is used to count the statistics. The formula for counting pairs is shown in Equation 1 below. The statistics to be computed are shown in Table 5. The linked pairs are all the pairs that SparkDWM linked and usually are the output from all the good clusters formed. On the other hand, equivalent pairs refer to the pairs of references found in the truth set file, and the system was expected to find and link those. True pair is the intersection between the pairs linked and those that were expected to be linked. With these base values, the precision, recall, and f-measure can be computed, as shown in Table 5. Precision is the number of true pairs divided by linked pairs, recall is the number of true pairs divided by the expected pair, and finally, f-measure refers to the harmonic mean between the precision and recall.


(1) $$\begin{array}{l} pair=\;\frac{\left(n^{*}\left(n-1\right)\right)}{2} \end{array}$$

**Table 5:** ER matrix calculation parameters.

**Matric**	**Formula**
Precision	True pairs/linked pairs
Recall	True pairs/expected pairs
F-measure	(2^*^P^*^R)/(P+R)

## 4 Experiment and results

In this section, we demonstrate SparkDWM using a set of commonly used data files used by the legacy DWM and expand on the system's capabilities with some publicly available benchmark ER data files. We also compare SparkDWM with HadoopDWM and report on the computational time of both systems.

### 4.1 Dataset

To prove that SparkDWM achieves the similar results as the legacy DWM we use a set of 18 synthetic datasets publicly made available and often used to test legacy DWM. These test samples were generated using the Synthetic Occupancy Generator (Talburt et al., 2009) program which infuses data quality errors into a given dataset in order to test the performance of ER systems. These 18 datasets contain customer names and address information, and the quality status spans from poor quality (with a P prefix) to good quality (with a G prefix), which are either in mixed layout (with a X prefix) or a single layout. Record headers found in the good-quality files include “recID”, “fname”, “lname”, “mname”, “address”, “city”, “state”, “zip”, “ssn”. Similarly, the record headers for the poor-quality files include “recID”, “name”, “address”, “city state zip”, “PO Box”, “POCity State Zip”, “SSN”, “DOB”. For system scalability testing purposes, we utilized publicly available datasets created by Köpcke et al. (2010). Three variations of these datasets were used including database affiliations which contains over 2,000 references, geographic settlement dataset containing approximately 3,000 references, and North Carolina (NC) voter's dataset. The NC voters dataset comprise of a 3.5 million, 7 million, and 203 million references.

### 4.2 Sparkdwm vs. legacy DWM

To compare the legacy DWM with SparkDWM, we used the same parameter file with optimal configuration values for both systems. These optimal values were determined using the PDP program (Anderson et al., 2023), which provides a set of starting parameters for the DWM. The number of records in each of the 18 sample files were small with S1G file being the least with about 50 records in it and the S6GeCo being the largest with 19,999 records. Given the smaller size of these files and the default HDFS block size being 128 MB, we utilized a single-node cluster. The cluster was had Spark with pre-built Hadoop 3.3, OpenJDK 8, and Python 3.10 installed. The host machine for the cluster was 64-bit Ubuntu 23.04 operating systems equipped with a 4-core i3 Intel 4th generation CPU at 3.10 GHz base speed and 8GB DDR3 RAM.

The result of the experiment is shown in Table 6 below. From Table 6, it can be seen that the SparkDWM system achieves the similar results as the legacy DWM. The two systems have the same Precision, Recall, and F-measure. For instance, with a good quality dataset such as S5G with 3,004 references, SparkDWM and legacy DWM had a precision of 0.9542, a recall of 0.9142, and an F-measure of 0.9338. For poor-quality datasets such as S9P, SparkDWM and legacy DWM had a precision of 0.8572, a recall of 0.6876, and an F-measure of 0.763. Similarly, for mixed layout datasets such as S15GX, SparkDWM, and legacy DWM had the precision of 0.9234, recall of 0.8684, and an F-measure of 0.8591, and for S18PX, SparkDWM, and legacy DWM had the precision of 0.8482, recall of 0.6609 and an F-measure of 0.7429. With all formats and layouts of datasets, SparkDWM achieves the similar result as legacy DWM, which is an indication that all the basic processes of the legacy DWM have been thoroughly refactored using PySpark's RDD.

**Table 6:** Comparison of ER metric results of SparkDWM and legacy DWM.

**Sample**	**Refs read**	**Quality**	**System**	**Precision**	**Recall**	**F-measure**
S1G	50	Good	Legacy DWM	1.0	1.0	1.0
			SparkDWM	1.0	1.0	1.0
S2G	100	Good	Legacy DWM	0.9231	1.0	0.96
			SparkDWM	0.9231	1.0	0.96
S3Rest	868	Good	Legacy DWM	0.9712	0.9018	0.9352
			SparkDWM	0.9712	0.9018	0.9352
S4G	1,912	Good	Legacy DWM	0.9649	0.9152	0.9394
			SparkDWM	0.9649	0.9152	0.9394
S5G	3,004	Good	Legacy DWM	0.9542	0.9142	0.9338
			SparkDWM	0.9542	0.9142	0.9338
S6GeCo	19,998	Good	Legacy DWM	0.9606	0.9769	0.9687
			SparkDWM	0.9606	0.9769	0.9687
S7GX	2,912	Good	Legacy DWM	0.9453	0.9067	0.9256
			SparkDWM	0.9453	0.9067	0.9256
S8P	1,000	Poor	Legacy DWM	0.8489	0.6677	0.7475
			SparkDWM	0.8489	0.6677	0.7475
S9P	1,000	Poor	Legacy DWM	0.8572	0.6876	0.7631
			SparkDWM	0.8572	0.6876	0.7631
S10PX	2,000	Poor	Legacy DWM	0.8845	0.6846	0.7718
			SparkDWM	0.8845	0.6846	0.7718
S11PX	3,999	Poor	Legacy DWM	0.8091	0.6788	0.7382
			SparkDWM	0.8091	0.6788	0.7382
S12PX	6,000	Poor	Legacy DWM	0.874	0.6849	0.768
			SparkDWM	0.874	0.6849	0.768
S13GX	2,000	Good	Legacy DWM	0.8843	0.8979	0.891
			SparkDWM	0.8843	0.8979	0.891
S14GX	5,000	Good	Legacy DWM	0.9186	0.8726	0.895
			SparkDWM	0.9186	0.8726	0.895
S15GX	10,000	Good	Legacy DWM	0.9234	0.8684	0.8951
			SparkDWM	0.9234	0.8684	0.8951
S16PX	2,000	Poor	Legacy DWM	0.8857	0.6904	0.7759
			SparkDWM	0.8857	0.6904	0.7759
S17PX	5,000	Poor	Legacy DWM	0.8699	0.6764	0.761
			SparkDWM	0.8699	0.6764	0.761
S18PX	10,000	Poor	Legacy DWM	0.8482	0.6609	0.7429
			SparkDWM	0.8482	0.6609	0.7429

### 4.3 Comparative analysis of SparkDWM and HadoopDWM

**Figure 4:**
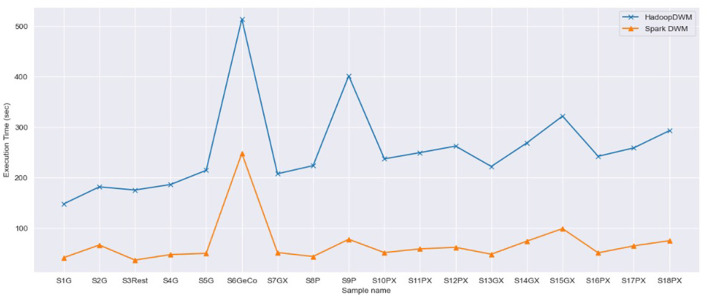
Comparison of SparkDWM execution time with HadoopDWM.

Next, we compare the performance of SparkDWM with the HadoopDWM (Hagan et al., 2024) system, a previously designed distributed DWM system using Hadoop MapReduce using the 18 synthetically generated datasets. Figure 4 shows the side-by-side comparison of the execution time of SparkDWM and HadoopDWM. From the graph, it can be observed that SparkDWM outperforms the Hadoop version of the DWM. The higher computational time of HadoopDWM was due to the reading and writing of intermediate data to and from disk, which is one of the bottlenecks of Hadoop MapReduce. In SparkDWM, the input data is read from HDFS once and all transformations in the execution process are stored in memory, hence the performance improvement.

### 4.4 Scalability of SparkDWM

To test the scalability of SparkDWM using larger data files, we used computational resources from the Arkansas High-Performance Computing Center's Pinnacle cluster. A maximum of 60 computational nodes were requested from the Pinnacle cluster. Each of the nodes was equipped with 2 8-core Intel(R) Xeon(R) Gold 6130 CPU @ 2.10GHz base speed, and a memory of 60 GB. Python version 10.12, Hadoop 3, Spark 3 with pre-built Hadoop, and Java 8 was installed on each computational node.

The dataset used for scalability testing includes the research author's affiliations dataset, geographic settlement dataset, and three versions of the North Carolina voter's dataset of sizes 3.5 million, 7 million, and 203 million. Table 7 shows the scalability and clustering behavior of SparkDWM using these publicly available ER benchmark datasets. The table includes the total reference count per sample, the program iterations, the number of references selected for reprocessing at each iteration step during the blocking phase, the number of pairs linked during the similarity comparison phase, the total clusters formed from each iteration, references in the cluster, the number of good clusters formed, and the total references in good clusters. SparkDWM iterates through the given dataset until all clusters formed are good and no other references are left during the blocking phase. At the end of each iteration, all unprocessed references and bad clusters formed (if any) are selected for reprocessing. For instance, in the first iteration of geographic settlement dataset, the total number of references in the formed clusters was 2,815; however, only 2,741 were good references in the 773 good clusters, leaving 74 bad clusters as shown in Table 7. The bad clusters were then merged with unprocessed references, totaling 313 for reprocessing in the second iteration. Similar scenarios apply to the affiliations and North Carolina datasets. In the affiliation's dataset, the 1st iteration had 2,260 references selected for reprocessing, the number of pairs linked was 6,530, total clusters formed was 222 with 1,668 references in those clusters, and out of the 222 clusters forms, all of them were good clusters as shown in Table 7. The number of references selected for reprocessing in the 2nd ietartion was 592 and because there were no linked pairs in that iteration, the SparkDWM hit an exit point and stopped.

**Table 7:** SparkDWM clustering statistics per program iteration.

**Data sample**	**Reference count**	**SparkDWM iteration**	**Selected refs to process**	**Linked pairs**	**Total clusters**	**Refs in clusters**	**Good clusters**	**Refs in good cluster**
Geographic settlement	3,054	1	3,054	3,357	796	2,815	773	2,741
		2	313	58	21	65	2	6
		3	307	50	18	55	2	5
		4	302	27	14	36	4	9
		5	293	7	5	12	1	2
		6	291	6	4	10	0	0
		7	291	4	3	7	0	0
		8	291	1	1	2	0	0
		9	291	0	-	-	-	-
Affiliations	2,260	1	2,260	6,530	222	1,668	222	1,668
		2	592	0	-	-	-	-
NC-Voters-3.5 mil	3,500,840	1	3,500,840	5,492	4,692	9,747	1,706	3,515
		2	3,497,325	3,449	2,974	6,203	0	0
		3	3,497,325	3,447	2,972	6,199	0	0
		4	3,497,325	3,443	2,970	6,194	0	0
NC-Voters-7 mil	7,001,680	1	7,001,680	1,065,973	836,693	1,754,634	823,312	1,670,126
NC-Voters-203 mil	203,048,721	1	203,048,721	299,549,642	165,061	9,865,452	165,061	9,865,452

We also compare the linking and clustering performance of SparkDWM with that of Famer. The result from the comparison is shown in Table 8. It can be observed from the table that with the affiliation's dataset, SparkDWM creates fewer linked pairs given optimal starting linking parameters from the PDP program compared with Famer. However, with similar optimal linking parameters, SparkDWM was over-linked when the Geographic Settlement file was processed, and in terms of clustering, SparkDWM over-clusters compared to what was recorded in Famer.

**Table 8:** Comparison of linking and clustering performance between SparkDWM and Famer.

**Sample**	**System**	**Total linked pairs**	**Total clusters**
Affiliations	SparkDWM	26, 844	814
	Famer	32, 816	330
Geographic Settlements	SparkDWM	4, 502	1,073
	Famer	4, 391	820

Table 9 shows the performance comparison of SparkDWM and HadoopDWM using dataset sizes ranging from 3.5 million to 203 million references. Each computational node used for these experiments has an 8-core CPU and 60 GB of memory. It is observed from the experiment that for 3.5 million entity references, the total job required five computational nodes, and SparkDWM took a total of 9 min to run. In contrast, it takes 36 min to run the same data in HadoopDWM. Similarly, for the 7 million references, only 5 computational nodes were required, and it took SparkDWM 15 min to run compared to 2 h in HadoopDWM. Again, for the 203 million references, 41 computational nodes and a total of 3 h 24 min were required to run successfully compared to 7 h 33 min in HadoopDWM. The higher computational time in HadoopDWM resulted from the reading and writing of data after each Mapper and Reducer step. SparkDWM, using a memory-based execution approach, achieves a better result than HadoopDWM in terms of execution time, as shown in Table 9.

**Table 9:** Performance comparison of SparkDWM vs. HadoopDWM for larger datasets.

**Sample name**	**Ref. count**	**Computational nodes**	**HadoopDWM execution time**	**SparkDWM execution time**
NC-Voters-3.5 mil	3,500,840	5	36 min	9 min
NC-Voters-7 mil	7,001,680	5	2 h 1 min	15 min
NC-Voters-203 mil	203,048,721	41	7 h, 33 min	3 h 24 min

## 5 Conclusion and future work

The use of distributed computing technologies for data processing has gained popularity since the advent of big data. These big data processing frameworks permit larger datasets to be partitioned into smaller chunks and processed in parallel. One of the most popular and widely used big data processing frameworks is Apache Spark. In this research, we introduced SparkDWM, which capitalizes on the scalability, in-memory computing, and highly distributed properties of PySpark to refactor a single-threaded design of a Data Washing Machine. We solved the out-of-memory problem of a DWM by using a single computer CPU cores and memory and scaled up the machine to be able to process larger datasets.

We tested the performance of SparkDWM with 18 synthetically generated name and address datasets and the results in Table 6 prove that SparkDWM gets similar results as the legacy DWM given optimal starting parameters from the PDP system. The 18 samples used were of varying formats and layouts, including poor quality, good quality, single layouts, and mixed layouts. The ER metrics from the experiment show that the file with the lowest reference count, S1G, has 50 references with precision, recall, and f-measure of 100%, respectively. On the other hand, the file with the highest number of references, S6GeCo, has approximately 19,999 references with a precision of 96.06%, recall of 97.69%, and f-measure of 96.87%.

SparkDWM was also compared with a previous system called HadoopDWM using the same 18 sample files. The results show that SparkDWM outperforms the Hadoop-based DWM in terms of execution time. It was observed that the higher computational time in HadoopDWM was caused by the extensive reading and writing of data to and from HDFS. Although Hadoop MapReduce is great for batch processing, the I/O overhead remains one of the main bottlenecks. PySpark stores intermediate RDD transformations in memory until an RDD action is invoked. This makes data processing in SparkDWM faster and more reliable than HDWM.

The scalability of SparkDWM was tested using a set of publicly available ER datasets. We tested the system using a dataset from a few thousand to 203 million references and provided the system statistics in Tables 7, 9. In Table 9, we showed the computational time of SparkDWM and HadoopDWM given the same computational resources. For the 3.5 million references, SparkDWM took 9 min, whereas HadoopDWM took 36 min. Similarly, for 203 million references, SparkDWM took 3 h 24 min, whereas HadoopDWM took 7 h 33 min. We also compared the linking and clustering performance of SparkDWM with another distributed ER system, Famer, and concluded that SparkDWM over-clusters references given optimal parameters. However, SparkDWM can form fewer links than Famer. The number of linked pairs is easily improved if the linking threshold “mu” is reduced or more references are selected for processing in the blocking phase using “beta”. More often than not, having more linked pairs does not guarantee good clusters. We, therefore, learn that although Famer creates more linked pairs and fewer clusters, SparkDWM can form more clusters even with fewer linked pairs.

Since the main goal of this research was to prove that all the basic processes in the legacy DWM have been followed and SparkDWM is able to process hundreds of millions of records, we only compared our system's performance with the legacy DWM and Famer. In future research, we intend to perform a side-by-side performance analysis of SparkDWM with other distributed ER systems such as Dedoop and SparkER. This can be done by combining different execution types such as tiny executors and fat executors and comparing the linking, clustering, ER matrix performance of all these systems. Using fat executors means assigning all CPU cores on each computational node was assigned to only 1 executor. This was as a result of a poor performance observed when we utilized tiny executors where there is 1 CPU core assigned per each executor on the cluster. On the other hand, using tiny executors mean assigning 1 CPU core per executor on the cluster. Given the shortcomings in both configuration approaches, we belief a combination of both configurations will produce a better result.

Again, in future research, we plan to incorporate the PDP system and SparkDWM, where the dataset will be read once for both systems, and the determination of starting parameters could be done simultaneously with SparkDWM in operation. Currently, using PDP to obtain the optimal starting parameters is a separate process from SparkDWM, and the PDP has to be run before SparkDWM. This means SparkDWM has to wait for PDP before processing data. This wait time can be eliminated if the PDP system is integrated into SparkDWM. This integration would help even further reduce computational time and eliminate the wait time for SparkDWM.

Lastly, in future research, we intend to improve the use of the Matrix Comparator as the linking method for SparkDWM. Although the Matrix Comparator was initially designed to handle small datasets, it has proven to be only a temporal solution for linking equivalent references in SparkDWM. Experiments even depict that the linking time is much higher when using the Matrix Comparator in SparkDWM compared to other data-intensive phases of the system, such as Blocking. Redesigning the Matrix Comparator will also improve linking performance of SparkDWM in terms of computational time.

## Data Availability

Publicly available datasets were analyzed in this study. This data can be found here: https://dbs.uni-leipzig.de/research/projects/object_matching/benchmark_datasets_for_entity_resolution.

## Footnotes

^1^ https://bitbucket.org/oysterer/distributed-dwm/src/master/

^2^ https://spark.apache.org/
